# Optimising DTwP-containing vaccine infant immunisation schedules (OptImms) — a protocol for two parallel, open-label, randomised controlled trials

**DOI:** 10.1186/s13063-023-07477-9

**Published:** 2023-07-21

**Authors:** Sarah Kelly, Xinxue Liu, Katherine Theiss-Nyland, Merryn Voysey, Sarah Murphy, Grace Li, Mary Nyantaro, Meeru Gurung, Sudha Basnet, Bhishma Pokhrel, Sanjeev M. Bijukchhe, Agnes Eordogh, Ben Gombe, Ayoub Kakande, Simon Kerridge, Geofrey Kimbugwe, Sylvia Kusemererwa, Lawrence Lubyayi, Henry Luzze, Olga Mazur, Yama F. Mujadidi, Shamim Nabukenya, Walter-Rodney Nagumo, Tryphena Nareeba, Rabiullah Noristani, Peter O’Reilly, Andrew Roberts, Ganesh Shah, Sonu Shrestha, Laxman P. Shrestha, Surya B. Thapa, Freddie M. Kibengo, Arun K. Sharma, Alison Elliott, Shrijana Shrestha, Andrew J. Pollard

**Affiliations:** 1 Oxford Vaccine Group, University of Oxford, Oxford, UK; 2 NIHR Oxford Biomedical Research Centre, Oxford, UK; 3 MRC/UVRI and LSHTM Uganda Research Unit, Entebbe, Uganda; 4 Patan Academy of Health Sciences, Lalitpur, Nepal; 5 Department of Paediatrics, Institute of Medicine, Tribhuvan University, Kathmandu, Nepal; 6 Ministry of Health, Kampala, Uganda

**Keywords:** Immunisation, Pertussis, Vaccination schedules, Global child health, EPI

## Abstract

**Background:**

Universal immunisation is the cornerstone of preventive medicine for children, The World Health Organisation (WHO) recommends diphtheria-tetanus-pertussis (DTP) vaccine administered at 6, 10 and 14 weeks of age as part of routine immunisation. However, globally, more than 17 unique DTP-containing vaccine schedules are in use. New vaccines for other diseases continue to be introduced into the infant immunisation schedule, resulting in an increasingly crowded schedule. The OptImms trial will assess whether antibody titres against pertussis and other antigens in childhood can be maintained whilst adjusting the current Expanded Programme on Immunisation (EPI) schedule to provide space for the introduction of new vaccines.

**Methods:**

The OptImms studies are two randomised, five-arm, non-inferiority clinical trials in Nepal and Uganda. Infants aged 6 weeks will be randomised to one of five primary vaccination schedules based on age at first DTwP-vaccination (6 versus 8 weeks of age), number of doses in the DTwP priming series (two versus three), and spacing of priming series vaccinations (4 versus 8 weeks). Additionally, participants will be randomised to receive their DTwP booster at 9 or 12 months of age. A further sub-study will compare the co-administration of typhoid vaccine with other routine vaccines at one year of age. The primary outcome is anti-pertussis toxin IgG antibodies measured at the time of the booster dose. Secondary outcomes include antibodies against other vaccine antigens in the primary schedule and their safety.

**Discussion:**

These data will provide key data to inform policy decisions on streamlining vaccination schedules in childhood.

**Trial registrations:**

ISRCTN12240140 (Nepa1, 7^th^ January 2021) and ISRCTN6036654 (Uganda, 17^th^ February 2021).

**Supplementary Information:**

The online version contains supplementary material available at 10.1186/s13063-023-07477-9.

## Background

Following the success of the smallpox eradication programme, the World Health Organisation Expanded Programme on Immunisation (WHO EPI) began in 1974, with the aim that all children in all countries could benefit from life-saving vaccines. At the time, the EPI included vaccination against six diseases: tuberculosis (BCG), diphtheria, tetanus and pertussis (DTP), measles and poliomyelitis. The list of recommended vaccines has since grown to include many new vaccines. Expansion of the immunisation schedules over the past two decades has significantly reduced death and morbidity among young children globally [1–3]. A substantial proportion of the protection is provided by herd immunity [4, 5]. In the future, new vaccines may be added to the EPI schedule, such as those for respiratory syncytial virus (RSV) and group B streptococcus.

Globally there are more than 17 unique DTP-containing schedules in use, with many countries aligning their schedules to regional norms. These include 2, 4, and 6 months (USA and the Americas); 2, 3, and 4 months (UK, western Europe, and central Asia); 6, 10, and 14 weeks (most of Africa and South Asia, recommended by WHO); 3, 5, and 12 months (Scandinavia and Italy); and 3, 4.5 and 6 months (Japan and China). Little evidence exists on the relative impact of these different schedules. Some evidence suggests that they may differ in key outcomes including immunogenicity, lasting protection, and acceptability by health workers and care-givers [6–8].

In 2009 the European Centre for Disease Control reviewed evidence for the optimal timing of DTP immunisation and due to the lack of available data, called for new studies to assess the differing schedules [9]. Four key research areas were highlighted: the optimal timing of the first DTP-containing vaccine, the number of doses in the priming series (two versus three), the interval between priming doses and lastly, the need for and timing of a booster dose to optimise immunity.

The current accelerated EPI schedule begins at 6 weeks of age and is designed to provide early protection against pertussis. However, there is a trade-off between earlier coverage and generation of strong immune responses due to the fact that weaker antibody responses to vaccination are seen in younger infants at this age [10]. Schedules which begin later and have longer intervals between doses are more immunogenic [11–13]. In reality, delivery of the EPI schedule on time is challenging for many countries; of 45 studied countries, the median delay for DTP1 was 2.4 weeks, and the median delay for DTP3 was 6.2 weeks [14].

Evidence from the trials of newer vaccines in infancy suggests that two doses given in a priming series may provide adequate protection. For example, pre-licensure trials of the 7-valent pneumococcal vaccine assessed 3 primary doses in the first 6 months of life and a 12-month booster [15]. However, this vaccine was successfully introduced to the UK in 2006 with a reduced 2-dose priming schedule at 2 and 4 months, and a 12-month booster, which was subsequently adjusted to a one dose priming schedule at 3 months of age with a booster at 12 months.

Nepal became the first Gavi-eligible country to adopt a variation on this schedule for the introduction of the 10-valent pneumococcal vaccine (6, 10 weeks and 9 months, with no further booster) reducing the number of injections, and total cost [16]. There is variability in the use of a booster for DTP, with some countries such as the UK, providing no booster until preschool age and others offering booster vaccinations at 12–18 months. The current accelerated WHO EPI schedule (6, 10, 14 weeks) does not include a booster dose. Currently, there is crowding of the vaccination schedule in early infancy due to the need to cover several differing antigens, with little room for new additions. However, it is likely that new childhood vaccines such as that for RSV are likely to require doses in early infancy if introduced [17].

There is a clear need for evidence to support optimisation of the current EPI schedule. The OptImms trials have been designed to identify an optimal immunisation schedule for infants by comparing the immunogenicity of 5 different priming immunisation schedules including the current WHO recommended schedule, and additional sub-studies comparing the impact of varied timing of booster doses, and co-administration of a typhoid conjugate vaccine (TCV) at one year of age.

## Methods

The OptImms trials are randomised, non-inferiority 5-arm clinical trials in Uganda and Nepal, using the WHO schedule as the reference schedule.

### Study sites

The Uganda site trial will take place at the Medical Research Council (MRC)/Uganda Virus Research Institute (UVRI) & London School of Hygiene and Tropical Medicine (LSHTM) Uganda Research Unit field site in the Greater Masaka area, Uganda. Masaka lies in the south-western Uganda region, approximately 120 kms from the capital city, Kampala. The population within the trial area is semi-urban, semi-rural. DTP1 coverage in Uganda was approximately 91% in 2019 with DTP3 coverage at 73% [18]. Infants will be enrolled at trial hubs located in three health facilities and clinics in the Masaka area. The Nepal site trial will take place in the two largest municipalities in the Kathmandu Valley: Lalitpur at Patan Academy of Health Sciences (PAHS), Patan Hospital and Kathmandu at the Institute of Medicine, Tribhuvan University Teaching Hospital (TUTH). The Kathmandu Valley is a large urban area with a high population density and is the largest urban area in Nepal. DTP1 coverage in Nepal, in 2019, was 96%, with DTP3 coverage at 93% [18, 19].

### Public engagement

Extensive community engagement has been taking place in both countries since 2019. The study teams will meet with local leaders, District and Municipality health teams, community members and in-country health and political leaders. The Uganda site has a well-established community advisory board who review the participant facing documents.

### Objectives

#### Primary objective

The primary objective of these studies is to identify a schedule (or schedules) that maintains early protection against pertussis prior to the booster dose and is equivalent to, or better, than the WHO EPI schedule.

#### Secondary objectives

1. Identify a schedule (or schedules) that maximises early protection against pertussis.
2. Identify a schedule (or schedules) that maximises protection against tetanus, diphtheria, pertussis, hepatitis B, *Haemophilus influenzae* type b (Hib), and pneumococcus post booster dose.
3. Identify a schedule (or schedules) that provides space in the immunisation programme for inclusion of new vaccines by minimising the number of diphtheria, tetanus, whole-cell pertussis, *Haemophilus influenzae* type b (Hib), hepatitis B virus (DTwP-HBV/Hib) doses (i.e. 2 priming doses rather than 3) whilst maintaining protection
4. Identify a schedule (or schedules) that maximises long-term protection at 24 months of age by vaccines for pneumococcus and tetanus, diphtheria, pertussis, hepatitis B, and *Haemophilus influenzae* type b (Hib).
5. Identify a schedule (or schedules) that maximises protection against tetanus, diphtheria, pertussis, hepatitis B, *Haemophilus influenzae* type b (Hib) and pneumococcus post-primary and pre-booster dose and polio (pre-booster only)
6. Assess the optimal timing of delivery of a booster dose of DTwP-Hib/HBV
7. Assess the effect of concomitant administration of typhoid conjugate vaccine with the Japanese encephalitis (JE)vaccine in Nepal and yellow fever (YF) vaccine in Uganda.

#### Exploratory objectives

1. Assess the optimal timing of measles-rubella vaccine administration

#### Safety objective

2. Assess the safety of alternative vaccination schedules in terms of reactogenicity post each vaccination dose

956 participants will be randomised to one of 5 primary schedules in each country:

- Arm 1: DTP at 6, 10 and 14 weeks (WHO EPI schedule)
- Arm 2: DTP at 6, 14 weeks + booster (modified WHO EPI)
- Arm 3: DTP at 2, 4 months + booster (OptImms schedule)
- Arm 4: DTP at 2, 3, 4 months (2–3–4 schedule)
- Arm 5: DTP at 2, 4, 6 months (2–4–6 schedule)

The chosen schedules (Table 1) compare how variation in timing, number of doses, dose interval and booster doses may affect immunogenicity and persistence of antibody generated in response to EPI vaccinations. Each schedule has been selected based on real-world use, globally, or immunogenicity data from previous studies: Arm 1 represents the WHO-recommended “accelerated” schedule and serves as the control arm for the purpose of statistical analyses. Arms 2 and 3 are two variations on the WHO 2 + 1 reduced priming schedule — one beginning at 6 weeks, and one with wider intervals between doses, beginning at 8 weeks. Arms 4 and 5 represent schedules used in the UK and the Americas, respectively.

**Table 1:**
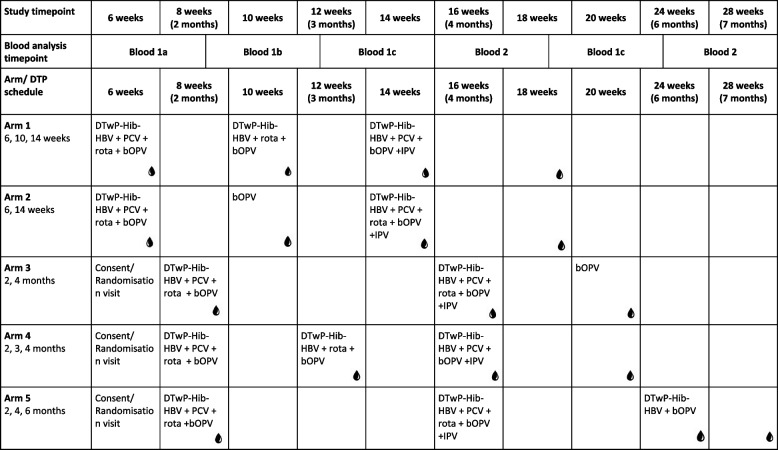
Main vaccine schedules for each primary vaccine arm and blood draw timepoints

In addition to randomisation to one of five main primary vaccination schedules, participants will be randomised to receive booster vaccines at one of two time points (9 months or 12 months) and in addition (1) concomitant TCV, (2) concomitant Japanese encephalitis (JE) (Nepal only) or YF (Uganda only), or (3) concomitant TCV with either JE (Nepal) or YF (Uganda).

The booster schedules will investigate the effect of the timing of the DTP-containing booster, as well as the immune response to the concomitant administration of TCV with other routine EPI vaccines (DTP, JE and/or YF) when administered together or separately.

### Randomisation

Participants will be electronically randomised to one of the 5 main primary vaccination arms in a ratio of 4:4:4:3:3, using block randomisation. This will take place within an eCRF hosted by REDCap (v11.1.8). For arms 1–4, a second randomisation will allocate participants to 1 of 4 booster groups, in a ratio of 3:1:1:1. For arm 5, participants will be allocated in a 1:1:1 ratio to booster groups 2–4 (see Table 2). Half of the study participants will be randomised to receive a DTP-containing booster at 9 months (Booster Group 1), together with the MR and PCV vaccinations already recommended by the WHO EPI at this timepoint. Participants in this booster group will then receive TCV vaccination at 10 months. The remaining half will be randomised to receive a DTP booster at 12 months of age, given either with both TCV and JE/YF, or one of these at 12 months and one at 13 months, to evaluate any effects of concomitant vaccine administration on immunogenicity.

**Table 2:**
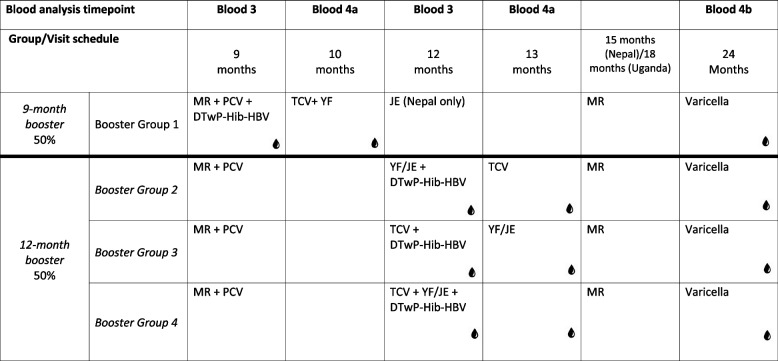
Booster group vaccine schedules and blood draw timepoints

To minimise the number of blood samples for each infant, participants will be randomised to receive 4 blood samples across the two-year follow-up period. The possible blood sample time points vary between the 5 main vaccination arms (Tables 1 and 2). All participants will have a blood sample taken 1-month post-priming series and pre-booster. Two additional blood samples will be taken: one during the priming series, and one during the booster series.

The following vaccine formulations to be administered in this trial will be the same across all sites and are outlined in Table 3 below. All other vaccines given in this trial will be in line with the vaccines currently used as part of the country’s immunisation programme.

**Table 3:** Vaccine formulations to be administered in OptImms

Manufacturer	Vaccine	Contents
Serum Institute of India	DTwP-HBV-Hib	Diphtheria toxoid ≥ 30 IU, Tetanus toxoid ≥ 40 IU B. Pertussis (whole cell) ≥ 4 IU, Hep B surface antigen (rDNA) 10mcg, Purified capsular polysaccharide of Hib (PRP) conjugated to tetanus toxoid (carrier protein) 10mcg
Bharat-Biotech	Typhoid conjugate	Purified Vi-capsular polysaccharide of *S. Typhi* Ty2 conjugated to Tetanus Toxoid 25 µg
Sanofi Pasteur	Yellow fever	Yellow fever virus1 17D-204 strain (live, attenuated) not less than 1000 IU produced in specified pathogen-free chick embryos

Trial arm allocation will be open-label for participants, clinical trial staff and statisticians, but laboratory staff will be blinded.

### Participants

Parents/guardians of healthy children aged 42–50 days will be approached when they present for their 6-week immunisations. All healthy children, living in the study area, will be eligible for enrolment in the study. Children will be excluded if born prematurely (< 37 weeks gestation), at a low birth weight (< 2.5 kg), or presenting with serious health concerns, as determined by study medical staff (see Appendix).

### Enrolment

At the enrolment visit, children will go through a pre-screening process, including confirmation of age and birth weight with an assessment of temperature. Written and verbal versions of participant information and informed consent documentation will be provided to parents/guardians by trial staff including clinicians, health care staff and counsellors. Impartial witnesses will be used in cases where parents/guardians are unable to read participant information leaflets. Trial staff will inform the participants the study arm to which they have been allocated including time of booster doses and blood draw visits. Participants will be given the opportunity to ask questions about their allocated study arm, to ensure full understanding of the schedule to which they have been randomised. They will be asked to provide written informed consent by means of a signature or thumbprint.

The participant will be considered enrolled once the consent form is signed, eligibility criteria are met and the participant has been randomised into a trial arm. They will receive an OptImms-specific child health card aligned to the format of the local child health cards currently in use in the study country. This will include the immunisation schedule for the participant’s allocated trial arm, infant growth charts, infant health advice, and space for non-study vaccinations to be recorded.

### Benefits to participants

Free routine medical care and treatment of infections will be provided to all participants during the course of the study. Participants may also receive benefits from the administration of additional study vaccines not currently in the Nepal/Uganda EPI schedule; Yellow fever vaccine (currently recommended in Uganda but not offered routinely), typhoid conjugate vaccine administered during the booster phase of the study, and varicella vaccine which is offered to all participants at 2 years of age. Travel costs will be reimbursed for all participants at each visit.

### Study visits and participant follow-up

At each study visit, routine and study vaccines will be administered and/or study blood draws performed as per the allocated arm. Topical anaesthetic cream will be used at each blood draw visit to minimise pain, discomfort and distress for participants and their parents.

Participants will also receive follow-up phone calls from the study team, to maintain contact with participants and their families and to help ensure participants stay on their designated study schedule.

If a parent/guardian decides that they wish to withdraw from the trial, catch-up immunisation will be arranged by the local teams to ensure participants have not been disadvantaged by their participation in the study and that they have received immunisations in line with the national EPI schedule in that country.

For participants receiving a specific antigen schedule outside of the WHO recommendations, and where immunological data are available for that participant, an additional dose of the relevant vaccine will be offered to children who have not mounted an immune response to the accepted threshold of protection, measured one month after the final dose of that vaccine. For children who meet these criteria, this additional vaccine will be offered after the immunological data have been analysed, at the end of the study.

### Laboratory assays

Assays for both countries will be carried out in the same laboratory. Serum will be used for assessing immune responses to each component of the vaccines included in the study. This will be measured by multiplex immuno-assay, enzyme-linked immunosorbent assay (ELISA), plaque reduction neutralisation tests (PRNT), or indirect fluorescent antibody tests as applicable (Table 4).

**Table 4:** Antigens and assays for primary and secondary immunogenicity outcomes

Antigen	Assay	Volume of serum required (µl)
Diphtheria toxin, tetanus toxin, pertussis (pertussis toxin, pertactin, filamentous hemagglutinin), Hib-PRP, hepatitis B surface antigen, polio types 1–3, pneumococcal antigen (all 10 serotypes)	Multiplexed immunoassay	150
Japanese encephalitis virus	Enzyme-linked immunosorbent assay	100
	Plaque reduction neutralisation titres/indirect fluorescent antibody assay	50
Yellow fever virus	Enzyme-linked immunosorbent assay	100
	Plaque reduction neutralisation titres	30–80
Typhoid Vi antigen	Enzyme-linked immunosorbent assay (IgG and IgA)	60–100

### Data management

Data will be collected via direct data entry, into the study REDCap database. Databases will be hosted on physically secure local servers within each study country, with data management oversight being led by the University of Oxford team in collaboration with local data teams. Study staff will be responsible for ensuring that participants’ confidentiality is maintained with identification only via a participant identifier on study documentation and databases. All study documentation in hard copy will be stored securely in line with Good Clinical Practice guidelines.

### Data analysis

The trial dataset will be made available to study statisticians. Primary outcome, anti-pertussis toxin IgG measured prior to the booster dose, will be assessed using a non-inferiority comparison. Primary analyses will be carried out by separately comparing Arms 2 and 3, to Arm 1. If the lower bound of the one-sided 95% confidence interval for the difference in log-geometric means for any group compared to Arm 1 excludes a standardised effect size of — − 0.35 (Cohen’s d) then non-inferiority will be deemed to have been demonstrated. In the primary analysis, we will present the results from both intention-to-treat and per-protocol analysis. Secondary and exploratory outcomes will either be analysed descriptively or using superiority comparisons unless specified otherwise in the statistical analysis plan. The trial will not directly evaluate whether there is an interaction between the primary vaccination series and vaccination with TCV and YF (Uganda) or JE (Nepal). There is no planned interim analysis and no formal stopping rules for this trial.

### Sample size

Based on recent publications on the trial vaccine DTwP-HBV-Hib (Serum Institute of India), we found that the geometric mean concentration (GMC) and standard deviation (SD) of anti-pertussis IgG vary between studies and there is no consensus on the threshold of protection [20–25]. The sample size calculation for this trial is based on a non-inferiority margin presented as a proportion change in units of standard deviation (SD), or Cohen’s D. Since the two main non-inferiority comparisons will be “Modified EPI” (Arm 2) and “OptImms” (Arm 3) vs “WHO EPI” (Arm 1), the type I error is set as 0.025. The assumptions for the power calculation are:

- Power of 90%
- Non-inferiority margin of 35% SD
- Type I error of one-sided 0.025

Table 5 shows the non-inferiority margin based on different SDs in the current publications of the trial vaccine.

**Table 5:** Non-inferiority margins based on varying standard deviations (SDs)

Standard deviation	Non-inferiority margin on Log_10_ scale
Standardised standard deviation (SD = 1) on the Log_10_ scale	− 0.35 (Cohen’s d)
Assuming an SD of 0.6 on the Log_10_ scale [20]	− 0.21 (equivalent to a GMR of 0.62)
Assuming an SD of 0.2 on the Log_10_ scale [23]	− 0.07 (equivalent to a GMR of 0.85)

A minimum of 782 children per country are required for a fully powered analysis of the primary outcome, therefore each country will recruit 956 children to allow for loss-to follow-up of approximately 15%.

### Monitoring and safety reporting

An independent, international data safety monitoring board (DSMB) will monitor both OptImms trials. All suspected pertussis cases and deaths will be reported within 24 h of the case being known to the trial team throughout the trial period. Reviews of all recorded safety data and trial progress by the DSMB will take place at least 3 times a year or at the request of the trial management team, on demand or at a frequency determined by the severity of reported adverse events. The DSMB, sponsor, NHRC, UVRI and NDA will be kept updated about significant protocol amendments.

Monitoring will be performed throughout the study by representatives of the CTU and sponsor according to the principles of ICH GCP. Data will be evaluated for compliance with the protocol and accuracy in relation to source documents. Following a risk-based monitoring plan, the monitors will verify that the clinical study is conducted and data are generated, documented and reported in compliance with the protocol, GCP and the applicable regulatory requirements.

Virtual meetings of the trial management group will take place weekly during the recruitment phase of the study, and fortnightly once recruitment is complete to discuss study progress, any issues identified during data management checks and monitoring visits or as a result of adverse event reporting. An investigator meeting will be held annually to discuss study progress and results. Attendees will include (but not be limited to) the trial management team, key members of the institution where the samples will be processed and funder representatives.

All parent/guardian-reported adverse events following vaccination, occurring within the first 7 days post-vaccination administration will be recorded. National reporting guidelines for Serious Adverse Events (SAEs) will be followed. All SAEs which occur within the first 30 days post-vaccination will be recorded and Serious Adverse Reactions (SARs) will be reported to the Chief and Principal Investigators and sponsor (University of Oxford) within 24 h of trial team becoming aware of the event, and local ethics committees in line with national reporting guidelines within 7 calendar days. Monitoring visits will be conducted at regular intervals throughout the trial in conjunction with the sponsor.

## Discussion

OptImms will provide new data that will for the first time allow head-to-head comparisons across the most commonly used schedules for EPI around the globe. The data will be a major contribution to the ongoing review of the EPI at the WHO, and the EPI rationalisation that is needed in the face of new vaccines that will be included in schedules in the near future (such as malaria). Policymakers will then be able to make evidence-based decisions on the optimal schedule for different settings. Both OptImms-Uganda (protocol version 3.0, November 2021) and OptImms-Nepal (protocol version 2.0, November 2021) commenced recruitment in Autumn 2021. Initial results from these two trials are anticipated in 2023. The results of this study will be promptly published in peer-reviewed open-access journals.

### Supplementary Information

**Additional file 1. **SPIRIT 2013 Checklist: Recommended items to address in a clinical trial protocol and related documents*.

**Additional file 2. **SPIRIT diagram for OPTIMMS studies.

## Data Availability

Not applicable as data collection is ongoing.

## Appendix

### Inclusion criteria

**Table:**

Criteria
Age of 42–50 days old at time of first visit
Generally healthy as determined by a medical history and examination
Resident in the Kathmandu Valley, Nepal study area and planning to remain in the study area for the 2 years of the study

### Exclusion criteria

**Table:**

Criteria
Born at less than 37 weeks’ gestation
Birth weight < 2.5 kg or a present weight of < 3 kg at 6 weeks of age, as determined by a medical professional
Prior receipt of any vaccination except polio, hepatitis B, or BCG
Planned administration of vaccines other than the study vaccines (with the exception of vaccines against rotavirus, hepatitis A and B, inactivated influenza and varicella, which can be administered 14 days or 4 weeks in the case of two live viral vaccines (with the exception of polio) before or after study vaccines; polio and measles/rubella vaccines as part of national campaigns; and BCG vaccines which will be administered when indicated by national programme)
Parents who plan to move out of the geographical study area
Concurrently participating in another clinical study, which includes blood draws or IMPs, at any time during the study period, in which the participant has been or will be exposed to an investigational or a non-investigational product (pharmaceutical product or device)
Any major congenital defects, serious chronic illness, significant disease, disorder, family history or diagnosis of an immunosuppressive condition, or medical treatments which, in the opinion of the Investigator, may either put the participants at risk because of participation in the study, or may influence the result of the study, or the participant’s ability to participate in the study. (HIV and sickle cell are not exclusionary diagnoses in and of themselves)
Use of any investigational or non-registered product (drug or vaccine) within 30 days preceding the vaccination, or planned use during the study period
Known allergy to any vaccine components

## Abbreviations

BCG: Bacillus Calmette Guerin vaccine (tuberculosis vaccine)
DSMB: Data safety monitoring board
DTP: Diphtheria, tetanus, pertussis
DTwP-Hib/HBV: Diphtheria, tetanus, whole-cell pertussis, *Haemophilus influenzae* type b (Hib), hepatitis B virus
ELISA: Enzyme-linked immunosorbent assay
EPI: Expanded Programme on Immunisation
IFA: Indirect fluorescent antibody
IOM: Institute of Medicine
ISF: Investigator site file
JE: Japanese Encephalitis
MR: Measles-Rubella
MRC/UVRI & LSHTM: Medical Research Council/Uganda Virus Research Institute & London School of Hygiene and Tropical Medicine
NDA: National Drug Authority
OxTREC: Oxford Tropical Research Ethics Committee
PAHS: Patan Academy of Health Sciences
PCV: Pneumococcal conjugate vaccine
PRNT: Plaque reduction neutralisation titres
RSV: Respiratory syncytial virus
SAE: Serious adverse event
SAR: Serious adverse reaction
TCV: Typhoid conjugate vaccine
TUTH: Tribhuvan University Teaching Hospital
UNCST: Uganda National Council for Science and Technology
UK: United Kingdom
WHO: World Health Organisation
YF: Yellow fever
