# Supplementary material for: Optimising DTwP-containing vaccine infant immunisation schedules (OptImms) — a protocol for two parallel, open-label, randomised controlled trials
Source: Trials. 2023 Jul 21;24:465. doi: 10.1186/s13063-023-07477-9 (PMC10360224; doi:10.1186/s13063-023-07477-9)
Supplement: Supplementary file 2 — Additional file 2. SPIRIT diagram for OPTIMMS studies. [file 13063_2023_7477_MOESM2_ESM.doc]

SPIRIT diagram for OPTIMMS studies

|  | **Enrolment** |  | | | | | | | | | | | | | | | |
| --- | --- | --- | --- | --- | --- | --- | --- | --- | --- | --- | --- | --- | --- | --- | --- | --- | --- |
| **TIMEPOINT (days of life)** | **D42-50** | ***6 wks*** | ***8***  ***wks*** | ***10 wks*** | ***3 mth*** | ***14 wks*** | ***4 mth*** | ***18 wks*** | ***20 wks*** | ***6 mth*** | ***7 mth*** | ***9 mth*** | ***10 mth*** | ***12 mth*** | ***13 mth*** | ***15 mth*** | ***2 yr*** |
| **ENROLMENT:** | X |  |  |  |  |  |  |  |  |  |  |  |  |  |  |  |  |
| **Eligibility screen** | X |  |  |  |  |  |  |  |  |  |  |  |  |  |  |  |  |
| **Informed consent** | X |  |  |  |  |  |  |  |  |  |  |  |  |  |  |  |  |
| **Height and weight** | X |  |  |  |  |  |  |  |  |  |  |  |  |  |  |  |  |
| ***Allocation*** | X |  |  |  |  |  |  |  |  |  |  |  |  |  |  |  |  |
| ***Provide Child Health Cards*** | X |  |  |  |  |  |  |  |  |  |  |  |  |  |  |  |  |
| **INTERVENTIONS:** |  |  |  |  |  |  |  |  |  |  |  |  |  |  |  |  |  |
| **Arm 1** |  | X |  | X |  | X |  |  |  |  |  |  |  |  |  |  |  |
| **Arm 2** |  | X |  | X |  | X |  |  |  |  |  |  |  |  |  |  |  |
| **Arm 3** |  |  | X |  |  |  | X |  | X |  |  |  |  |  |  |  |  |
| **Arm 4** |  |  | X |  | X |  | X |  |  |  |  |  |  |  |  |  |  |
| **Arm 5** |  |  | X |  |  |  | X |  |  | X |  |  |  |  |  |  |  |
| **Booster Group 1** |  |  |  |  |  |  |  |  |  |  |  | X | X | X |  | X | X |
| **Booster Group 2** |  |  |  |  |  |  |  |  |  |  |  | X |  | X | X | X | X |
| **Booster Group 3** |  |  |  |  |  |  |  |  |  |  |  | X |  | X | X | X | X |
| **Booster Group 4** |  |  |  |  |  |  |  |  |  |  |  | X |  | X |  | X | X |
| **ASSESSMENTS:** |  |  |  |  |  |  |  |  |  |  |  |  |  |  |  |  |  |
| ***Temperature at vaccination visits*** | X | X | X | X | X | X | X |  | X | X |  | X | X | X | X | X | X |
| ***Medical history*** | X | 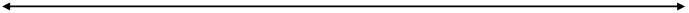 |  |  |  |  |  |  |  |  |  |  |  |  |  |  |  |
| ***Blood tests as per randomisation*** |  |  |  |  |  |  |  |  |  |  |  |  |  |  |  |  |  |
|  |  |  |  |  |  |  |  |  |  |  |  |  |  |  |  |  |  |
